# Conserved and Diversified Mechanism of Autophagy between Plants and Animals upon Various Stresses

**DOI:** 10.3390/antiox10111736

**Published:** 2021-10-29

**Authors:** Naveed Ur Rehman, Peichun Zeng, Zulong Mo, Shaoying Guo, Yunfeng Liu, Yifeng Huang, Qingjun Xie

**Affiliations:** 1 State Key Laboratory for Conservation and Utilization of Subtropical Agro-Bioresources, Guangdong Provincial Key Laboratory of Plant Molecular Breeding, South China Agricultural University, Guangzhou 510642, China; naveed.urrehman@yahoo.com (N.U.R.); zpc2019@stu.scau.edu.cn (P.Z.); gustaf@stu.scau.edu.cn (Z.M.); syguo6688@163.com (S.G.); 2 State Key Laboratory for Conservation and Utilization of Subtropical Agro-Bioresources, College of Life Sciences and Technology, Guangxi University, Nanning 530004, China; yunfengliu_bio@126.com; 3 Institute of Crop and Nuclear Technology Utilization, Zhejiang Academy of Agricultural Science, Hangzhou 310001, China

**Keywords:** autophagy, degradation, vacuole, autophagosomes, autophagy-related protein

## Abstract

Autophagy is a highly conserved degradation mechanism in eukaryotes, executing the breakdown of unwanted cell components and subsequent recycling of cellular material for stress relief through vacuole-dependence in plants and yeast while it is lysosome-dependent in animal manner. Upon stress, different types of autophagy are stimulated to operate certain biological processes by employing specific selective autophagy receptors (SARs), which hijack the cargo proteins or organelles to the autophagy machinery for subsequent destruction in the vacuole/lysosome. Despite recent advances in autophagy, the conserved and diversified mechanism of autophagy in response to various stresses between plants and animals still remain a mystery. In this review, we intend to summarize and discuss the characterization of the SARs and their corresponding processes, expectantly advancing the scope and perspective of the evolutionary fate of autophagy between plants and animals.

## 1. Introduction

To overcome the stress challenges, eukaryotes have evolved all sorts of sophisticated mechanisms to deal with the adverse effects of stress. Among them, autophagy (meaning “self-eating”) is one of the most robust mechanisms used to manage cytoplasmic material, such as nucleic acid aggregates, protein complexes, lipid bodies, and damaged organelles [1], ultimately resulting in the turnover of cellular components in the lytic organelle (vacuole in plants and yeast and lysosome in animals) [2]. Autophagy can digest certain cell components selectively or non-selectively by degrading bulk cytoplasm. In each case, the cellular components and macromolecules are encircled by a double membrane vesicle, termed an autophagosome, which merges with the vacuole for degradation and then recycles cellular components [3]. The biogenesis of the autophagosome is generally derived from the endoplasmic reticulum (ER) by generating a double membrane envelope called phagophore. However, there is still another notion that autophagosome may be produced by other membranes [4]. Owing to the discovery of *AuTophagy-related Genes* (*ATG*), the regulatory route of autophagic machinery has been well documented among various species based on the conservation of ATG proteins [5]. Briefly, initiation, nucleation, elongation, and fusion/degradation are the four phases of the autophagic process [6,7].

Autophagy is a quality control process in plants that fine-tunes the circulation of cell components. During development, it also plays a role in aging, pollen maturation, and programmed cell death (PCD) [8]. Moreover, autophagy occurs at low-intensity under normal conditions; however, it is drastically intensified when confronts with various abiotic and biotic stresses (e.g., carbon or nitrogen deficiency, salt, drought, temperature, reactive oxygen species, or infections) [9]. On the other hand, autophagy plays a crucial part in mammals’ appropriate growth and development, beginning with embryogenesis [10]. It is critical for good health since its proper functioning inhibits the onset of various diseases, including cancer, liver, muscle, and heart problems, neurological disorders (such as Huntington’s disease), inflammation, pathogen infections, and aging [11].

In plants, there are three types of autophagy mechanisms: microautophagy, macroautophagy, and mega-autophagy [12]. Microautophagy is a pattern in which the vacuole membrane invagination directly packages target substrates in the cytoplasm, and the bundled substrates are then degraded for cyclic use. In plants, macroautophagy is characterized by the presence of a large autophagic vacuole with a double-membrane structure that is utilized to package and transport toxic cytoplasmic components for degradation [13]. Mega-autophagy is only found in plants and occurs concomitantly with developmental programmed cell death (PCD). Throughout mega-autophagy, large amounts of hydrolases are released into the cytoplasm from the vacuole, resulting in large-scale degradation of cellular components including cytoplasm, all organelles, the plasma membrane, and part of the cell wall [12,13]. Unlike microautophagy and macroautophagy that recycle macromolecular constituents back to the cytosol from the vacuole, mega-autophagy is an extreme form of massive degradation leading to cell death [14].

In mammalian cells, the lysosomal membrane invaginations/protrusions are employed to collect cargo during microautophagy [15]. Microautophagosomes are formed close to the vacuole, while macroautophagosomes occur far from it [2,12]. Moreover, chaperone-mediated autophagy (CMA) differs from microautophagy as it requires chaperones to recognize cargo proteins where these substrates are independently unfolded and translocated via the lysosomal membrane [16]. Unlike microautophagy and CMA, macroautophagy comprises of the sequestration of cargo away from the lysosome and, subsequently, de novo synthesis of autophagosomes is employed to sequester the cargo and carry it to the lysosome [17]. In this review, we are in particular attempting to advance the current knowledge of autophagy and discuss the distinct and conserved mechanism of autophagy between plants and animals.

## 2. Mechanism of Autophagy in Plants and Animals

Although autophagosomes were initially discovered in mammalian cells in the 1950s [18], the molecular principles of autophagy were originally explored in yeast and subsequently expanded to animal and plant cells by the characterization of ATG proteins [13,19,20]. To date, the ATGs driving autophagy have been thoroughly understood in terms of induction, cargo recognition, phagophore generation, development, autophagosome fusion, and degradation [21]. In yeast, more than 40 *ATG* genes have been isolated, leading to the identification of many *ATG* homologs in mammals and plants (Table 1) [9]. In plants, such as Arabidopsis (*Arabidopsis thaliana*), roughly 40 ATGs have been discovered according to the protein similarity to yeast ATGs [22]. These ATG proteins are mostly clustered into four functional categories: (1) the ATG1/ATG13 kinase complex, which triggers the formation of autophagosome under nutrient deprivation; (2) the autophagy-specific class III phosphatidylinositol (PI) 3 kinase complex; (3) the ATG8/ATG12 ubiquitin-like conjugation systems that act in phagophore expansion; and (4) the ATG9 complex, which stimulates phagophore expansion [1,23].

**Figure 1:**
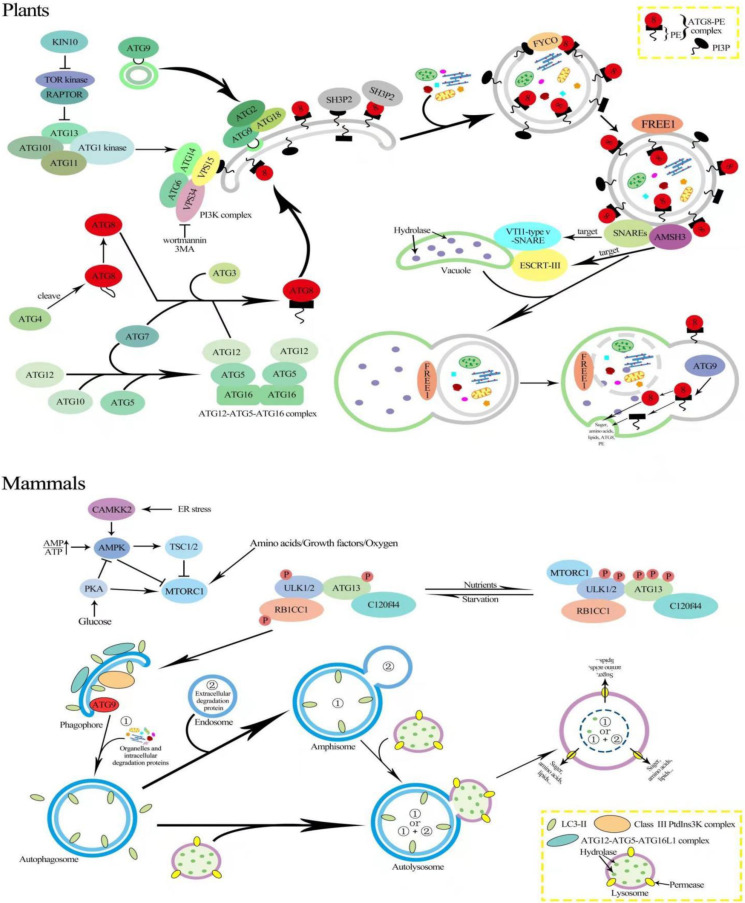
Schematic illustration of autophagy regulation in plants and animals. Autophagy is activated by inhibiting TOR and is blocked when TOR is overexpressed. Autophagy is triggered by the formation of an active complex between ATG13, ATG1, ATG11, and ATG101, as well as ATG11 and ATG101, which activates autophagy. Autophagosome development comprises membrane delivery, nucleation, expansion, and closure of the phagophore. ATG9 is employed in the transport of lipids to the expanding phagophore, together with ATG2 and ATG18. PI3P decoration is generated by the VPS34 lipid kinase complex, which is followed by ATG8 conjugation to PE. Initially, ATG8 is matured by ATG4 cleaving of its C-terminal and conjugating it to PE by E2-like ATG3 and the E3-like ATG12–ATG5–ATG16 complex. For phagophore expansion, ATG8–PE binds to the autophagosomal membrane. Sealed ATG8- and PI3P-decorated autophagosomes are transported to the vacuole with the help of FYCO (FYVE and coiled-coil domain-containing) proteins that bind the autophagosome to the microtubule transport machinery. With the aid of ARP2/3 (NAP1), ESCRT (CFS1, CHMP1, FREE1, and VPS2.1), and exocyst (EXO70B1) components, SNARE-mediated fusion of autophagosomes with the tonoplast releases autophagic bodies into the vacuole. Following that, vacuolar hydrolases degrade the vesicles. Model of Ulk1 regulation by AMPK and mTORC1 in response to glucose signals. Left: when glucose is sufficient, AMPK is inactive and mTORC1 is active. The active mTORC1 phosphorylates Ulk1 on Ser 757 to prevent Ulk1 interaction with and activation by AMPK. When cellular energy level is limited, AMPK is activated and mTORC1 is inhibited by AMPK through the phosphorylation of TSC2 and Raptor. The induction complex consists of ULK1/2, ATG13, RB1CC1, and C12orf44. Under nutrient-rich conditions, MTORC1 associates with the complex and inactivates ULK1/2 and ATG13 through phosphorylation. During starvation, MTORC1 dissociates from the complex, and ATG13 and ULK1/2 become partially dephosphorylated by yet-unidentified phosphatases, allowing the complex to induce macroautophagy. RB1CC1/FIP200 and C12orf44/ATG101 are also associated with the induction complex and are essential for macroautophagy. RB1CC1/FIP200 may be the ortholog of yeast Atg17, whereas the function of C12orf44/ATG101 is not known. A signal transduction event regulated by the TOR kinase leads to the following: (1) the induction of autophagy—a membrane from an unknown source sequesters cytosol and/or organelles resulting in the formation of a double-membrane vesicle termed an autophagosome; (2) on completion—the autophagosome docks with the lysosome or vacuole. Fusion of the autophagosome outer membrane with the vacuole releases the inner vesicle into the vacuole lumen. The inner vesicle is termed an autophagic body. Breakdown within the vacuole allows the recycling of the degraded autophagic body and its hydrolyzed cargo (amino acids, fatty acids, sugars, and nucleotides).

Two evolutionarily conserved protein kinase complexes, Target of Rapamycin (TOR) and Sucrose nonfermenting-1-Related protein Kinase 1 (SnRK1), compete for autophagy initiation [54]. TOR inhibits the conserved ATG1/ATG13 kinase activity, which is a negative regulator of autophagy (Figure 1) [26]. The TOR complex in Arabidopsis is made up of three main components: the TOR serine/threonine kinase [55], the regulatory-associated protein of TOR (RAPTOR) that supplies the substrates by TOR for phosphorylation [56,57], and the complex stabilizer LST8 [58]. TOR is widely expressed in actively growing tissues of Arabidopsis, such as endosperm, meristems, and embryos [55]. The reduced TOR expression, for example, results in reduced root growth, while overexpressing phenotypes show increased root growth [56]. TOR is rapidly activated under nutrient-rich conditions to accelerate development in sink tissues, in particular by Glc (glucose) after imported sucrose.

The protein kinase mechanistic target of rapamycin complex 1 (mTORC1/TORC1), which functions upstream of autophagy, includes mTOR, the regulatory associated protein of mTOR (Raptor), mammalian lethal with Sec13 protein 8 (mLst8/Lst8), proline-rich AKT substrate 40 kDa (PRAS40), and DEP domain-containing mTOR interacting protein (Deptor) [59]. Both growth factors and nutrition activate mTORC1 in the lysosome, which stimulates the translation regulating factors such as the ribosomal protein S6 kinase and the eukaryotic initiation factor 4E binding protein. Meanwhile, autophagy is suppressed by mTORC1 via phosphorylation of the ULK-complex [59]. Under glucose deficiency, AMPK directly senses the increase in the AMP:ATP ratio, leading to its activation [60]. Additionally, in response to glucose deprivation, AMPK suppresses mTORC1 by phosphorylating and activating the mTOR negative regulator tuberous sclerosis complex 2 (TSC2) (Figure 1) [61]. When nutrition levels are deprived, mTORC1 is repressed, and autophagy begins with ULK complex activation, the production of PI3KC3-mediated PI(3)P at the early autophagosomal membrane, the ATG12 complex, and the conjugation of the ATG8 family protein to the membrane lipid phosphatidylethanolamine (PE) [59].

In mammalian cells, the TOR complex suppresses ATG13–ULK1 interaction by phosphorylating ATG13, thus reducing autophagy, while AMPK stimulates autophagy by directly phosphorylating ULK1 (Figure 1) [62]. Notably, it is unclear if SnRK1/AMPK and/or TOR can directly phosphorylate ATG1 in plants, necessitating additional research [26,38]. Interestingly, even in plants overexpressing SnRK1 during hostile conditions, constitutive TOR expression inhibited autophagy, demonstrating that in both animals and plants, TOR that acts downstream of SnRK1/AMPK is crucial for autophagy induction [63]. Additionally, the overexpression of catalytic subunit of SnRK1 (KIN10) increases ATG1 phosphorylation in *Arabidopsis*, and the SnRK1–ATG1 interaction appears to exist in all plant tissues [38]. TOR is active in Arabidopsis and hyper-phosphorylates ATG13 under normal circumstances because highly phosphorylated ATG13 has a poor binding capacity for ATG1 so that the ATG1 activity is low and autophagy levels are maintained at baseline. In *Arabidopsis*, ATG1, ATG13, ATG11, and ATG101 form an active complex to stimulate autophagy (Figure 1) [26,64]. However, whether the ATG1–ATG13 complex is controlled by nutritional availability still remains a point of contention. In *Arabidopsis* membrane delivery, the nucleation, expansion, and closure of phagophores are all stimulated when the ATG1–ATG13 complex is activated [13,65]. ATG9 is involved in the development of the separation membrane at the phagophore assembly site (PAS) as well as in the supply of lipids to the growing phagophore, together with ATG2 and ATG18 (Figure 1) [4]. *Atg9* mutants in yeast and mammals do not generate autophagosomes, while in *Arabidopsis*, ATG9 deletion leads to the expansion of autophagosome-related tubules associated with the ER [4,66,67]. Furthermore, the sequence of *AtATG9* has little in common with that of yeast or humans [68], implying that *AtATG9* can work in plant-specific ways during the production of autophagosomes.

Autophagosome expansion and vesicle closure are aided by ATG8–PE, which is found in both the inner and outer autophagosome membranes [69,70]. In *Arabidopsis*, SH3P2 (SH3 domain-containing protein 2), a membrane-associated protein, translocates the PAS (phagophore assembly site) during autophagy (Figure 1) [71]. In addition to interacting with ATG8, SH3P2 also connects with PI3P and is involved in membrane elongation and autophagosome closure via the PI3K complex [71]. To ensure the movement of autophagosomes through the microtubules’ plus end, on the outer autophagosome membrane, LC3/ATG8 and PI3P bind with FYCO1 (FYVE and coiled-coil domain-containing 1) on the inner autophagosome (Figure 1) [72]. Moreover, co-sedimentation and co-localization tests in *Arabidopsis* revealed that ATG8 can bind to microtubules in vivo, implying that microtubules are involved in autophagosome migration to the vacuole [73].

In mammalian cells, autophagosomes go through a maturation process that includes PI(3)P turnover and the removal of ATG8 proteins by ATG4 proteases, as well as the recruitment of fusion machinery such as RAB7, the homotypic vacuole fusion and protein sorting (HOPS) tethering complex, and SNARES [74].

Unlike yeast, *Arabidopsis* possesses nine *ATG8* (*ATG8a*–*ATG8i*) homologs, two *ATG4* (*ATG4a* and *ATG4b*) homologs, and two *ATG12* (*ATG12a* and *ATG12b*) homologs [75,76]. The expression patterns of the *Arabidopsis ATG8* genes are tissue-specific, indicating that they may have diverse roles [77]. The ATG4s in Arabidopsis can cleave the C-terminus of ATG8, similar to their yeast counterparts. Furthermore, the *atg4a atg4b* double mutant exhibits autophagy defects, as shown by early senescence and lower silique synthesis, implying that ATG4s are required for plant growth [78]. The *atg12a atg12b* double mutant in *Arabidopsis* exhibits early senescence, food starvation sensitivity, and the absence of autophagic bodies, while the single mutants of *atg12a* and *atg12b* do not show, presenting functional redundancy. The ATG12–ATG5 conjugate accumulation was reduced in single mutants of *atg12a* or *atg12b* in which ATG8–PEs were not found, demonstrating that the ATG12–ATG5 binding is compulsory for ATG8–PE conjugation [79]. Mutations in plant ATG5, ATG7, or ATG10 result in hypersensitivity to nitrogen and carbon deficiency [79]. Likewise, *atg12*, *atg5*, and *atg10* mutants are unable to generate autophagic bodies in the vacuole [80].

Regarding the fusion of autophagosomes to the vacuole, several components have been implicated. For example, it was reported that SNAREs (soluble NSF attachment protein receptors) are required for accurate autophagosome targeting to the vacuole [81]. In *Arabidopsis*, the absence of VTI12, a VTI1-type v-SNARE (vesicle SNARE) on the target membrane, prevents autophagosomes from entering the vacuole under nutritional stresses, indicating that VTI12 is important for the fusion of the autophagosome [81]. AMSH3 (associated molecule with the STAM3 SH3 domain) is required for autophagosome trafficking to the vacuole in *Arabidopsis* and interacts with the ESCRT-III subunit VPS2.1 (vacuolar protein sorting 2.1) (Figure 1) [82]. Notably, in *Arabidopsis*, the plant-specific ESCRT component FREE1 (FYVE domain protein necessary for endosomal sorting 1) was discovered to interact with SH3P2 and to regulate the fusion of autophagosomes and vacuoles [71,83]. Furthermore, the interior vesicle, known as the autophagic body, is discharged into the vacuole when the autophagosome and vacuole are united and destroyed by a sequence of resident hydrolases [13]. The ATG8–PE linked to the inner autophagosome membrane is degraded into the vacuole, but ATG4 cleaves the ATG8–PE attached to the outside of autophagosome membrane, freeing ATG8 from PE and allowing it to be recycled [78].

In mammals cell, after lysosome fusion, lysosomal enzymes degrade the inner membrane of the autophagosome and its contents, and amino acids along with sugars are effluxed out of the lysosome by specific transporters, comprising of sugar efflux Spinster (SPNS), which is essential for degradation, autolysosome reformation, and the reactivation of mTORC1 [84].

## 3. Organelles Selective Autophagy

Organelle autophagy is essential for maintaining cellular homeostasis by preserving the integrity and quantity of organelles in changing environments and pressures. The specific selectivity of organelles by autophagy is governed by ATG8 interactions with specific autophagic receptors (termed SARs) with an ATG8-interacting motif (AIM) [85,86,87], resulting in different types of autophagy in regulating relevant biological processes.

### 3.1. Aggrephagy

**Figure 2:**
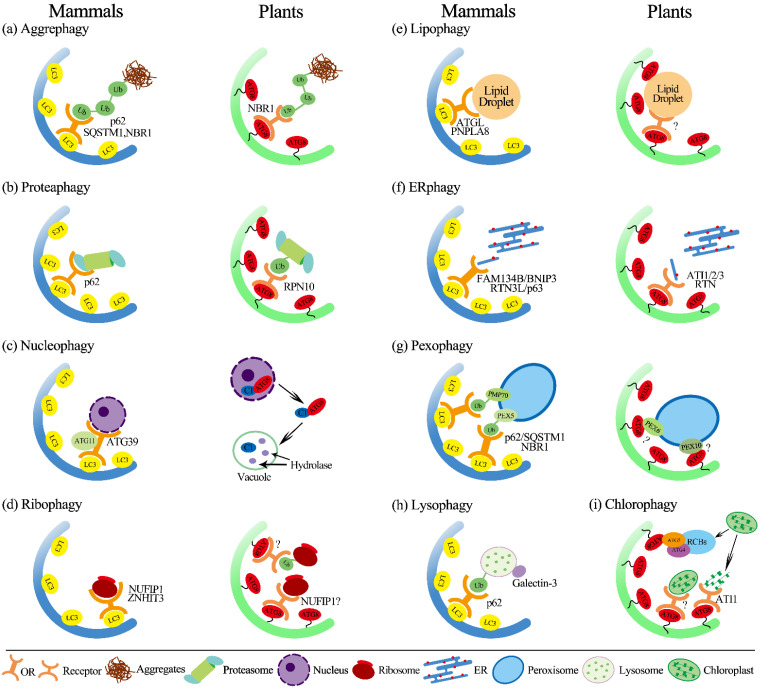
Schematic representation of several mechanisms of selective autophagy in plants and animals. The degradation autophagic pathways for cell organelles and aggregates are shown and distinct features of each are highlighted. (**a**) Aggrephagy. Degradation of intracellular protein aggregates that form naturally or as a result of abiotic stresses that cause protein folding. Aggrephagy is activated by aggregate ubiquitylation and autophagy-binding receptors, such as NBR1 in plants and p62/NBR1 in animals. (**b**) Proteaphagy. Degradation of proteasomes occurs in response to proteasome inactivation or nitrogen starvation. Proteaphagy is triggered by p62 in animals and RPN10 in plants and translocates it to the cytoplasm for degradation (**c**) Nucleophagy. Atg39 interacts with cargo receptor Atg11 through Atg11 binding region in animals and in plants ATG8 interacts with C1 and transports it to the cytoplasm from the nucleus. (**d**) Ribophagy. A ribophagy receptor NUFIP1 is essential for the selective degradation of ribosomes in animals and plants. (**e**) Lipophagy. PNPLA8 is required to produce autophagosomes during the lipophagy process in mammals while, in plants, no receptors have been identified so far. (**f**) Reticulophagy. The IRE1b stress sensor is required for endoplasmic reticulum degradation, which happens in response to an accumulation of unfolded proteins during ER stress. The reticulon homology domain (RTN) containing the family of reticulophagy receptors has been identified in mammals and yeast, but not in plants. ATI1 and ATI2 were the first ER-phagy receptors discovered in plants, and FAM134B, BNIP3, RTN3, and p63 have been identified as receptors in animals that translocate it to the cytoplasm for degradation. (**g**) Pexophagy. Pexophagy activates in response to ROS by phosphorylating PEX5 and PMP70 leading to ubiquitination recognized by p62, targeting peroxisomes for pexophagy. No pexophagy receptors have yet been described in plants, although the LON2 chaperone likely plays a role in peroxisome stress sensing, whereas PEX6 and PEX10 interact with ATG8. (**h**) Lysophagy. Removal of injured lysosome via concentrated recruiting of galectin-3 and LC3 onto lysosomal membranes, as these proteins are presumably recognized by p62/SQSTM1 and targeted for degradation via autophagy. (**i**) Chlorophagy. Chloroplasts are degraded in a variety of ways, including piecemeal degradation of stromal fragments in Rubisco-containing bodies (RCBs) during senescence or nutrient starvation, which may be mediated by ESCRT components such as CHMP1; the engulfment of whole chloroplasts in response to oxidative damage, which may be mediated by PUB4-dependent ubiquitylation; and the formation of ATI1/2 bodies.

Selective autophagy can also degrade nonfunctional proteins as aggregates, a process known as aggrephagy, with ubiquitin chains serving as a signal for degradation [88]. Aggrephagy receptors Cue5 in yeast and p62/SEQUESTOSOME 1 (SQSTM1) and Neighbor of BRCA 1 (NBR1) in mammals bind to ATG8 via the ubiquitin-binding domain (Figure 2) [89,90]. Plants have been shown to have a homolog of NBR1, an N-terminal PB1 (Phox and Bem1p) domain that binds to ubiquitin and ATG8 simultaneously, implying that aggrephagy mechanisms in yeast, plants, and mammals are similar (Figure 2a) [91]. NBR1 mutation causes an accumulation of ubiquitylated insoluble proteins in *Arabidopsis* during heat stress [92]. Furthermore, heat stress can drive NBR1 and ATG8 to bind with the aggregatic cytoplasmic protein, demonstrating that the plant aggrephagy receptor NBR1 is important in the regulation of proteostasis [93].

### 3.2. Proteaphagy

The eukaryotic proteasome contains the regulatory particle (RP), which is responsible for the recognition and unfolding of substrates, and the core particle (CP) for degradation [94]. Autophagy targets proteasomes in *Arabidopsis,* and it was previously confirmed that Arabidopsis RPN10 acts as a selective autophagy receptor and targets inactive 26S proteasomes by concurrent interactions with ubiquitylated proteasome subunits/targets and lipidated ATG8 lining, the enveloping autophagic membranes [95]. Previously, it was concluded that nitrogen deprivation induces autophagy in both proteasome subunits and is reliant on the lipidation of Atg8 via Atg7 and Atg10 [87]. Proteaphagy was increased in plants treated with the proteasome inhibitor MG132, whereas bulk autophagy remained unaltered, as determined by the lysosomal cleavage of GFP-Atg8. Notably, RPN10, a component of the RP which is essential for identifying ubiquitinated substrates, is required for proteaphagy (Figure 2b) [87,96]. RPN10 is a cytoplasmic protein that is not integrated into the proteasome, unlike other proteasomal proteins [87]. The binding motifs and sequence of RPN10 are substantially conserved among plants while neither the yeast nor human (PSMD4 in humans) homologs of Rpn10 have been confirmed to have any effect on proteaphagy or Atg8 [87,97]. Unlike yeast and plants, the animal proteasome becomes ubiquitinated upon starvation of amino acids, which is essential for its degradation by autophagy [13,87,98]. In animals, p62 can regulate the autophagosomal engagement of proteasomes by acting as a specific proteaphagy receptor (Figure 2b). Furthermore, autophagy receptor p62/SQSTM1, associated with numerous types of selective autophagy, recognizes the ubiquitin-modified proteasome [99]. These studies indicate that p62 is a key player in regulating the balance between proteasomal function and lysosomal degradation. Overall, it appears that proteaphagy occurs in a wide range of organisms, while molecular details vary that require further investigation.

### 3.3. Nucleophagy

The nucleus, similar to the eukaryotic cell primary organelle, is responsible for regulating gene expression and maintaining genomic integrity. When cells are stressed, they need a way to dispose of unwanted nuclear proteins and components. The mechanisms of nucleophagy are evolutionarily conserved catabolic processes which target various nuclear components such as the nuclear envelope, RNA, and DNA through a series of processes including nuclear sensing, nuclear export, and autophagic degradation in the cytoplasm [100]. For a long time, however, there was no proof that nucleophagy occurs in plants. The increase in the geminivirus nuclear protein C1 triggers autophagy, according to new research. Through the nuclear export-dependent process mediated by exportin 1 (XPO1), C1 is transported to the cytoplasm from the nucleus by interacting with ATG8h (NbATG8h or SlATG8h), one of several autophagy proteins, and when the AIM in C1 is mutated, it loses its ability to interact with ATG8 (Figure 2c) [101]. C1 degradation is prevented by inhibitors of autophagy and the removal of ATG5, ATG7, and ATG8h proteins [101]. In plants, this was the first time that autophagy was discovered to be involved in the breakdown of nuclear proteins.

Furthermore, a transcription factor BRI1-EMS Suppressor 1 (BES1), which controls brassinosteroid signaling, is ubiquitinated and interacts with DSK2A, leading to degradation in a DSK2 and core ATG-dependent way. DSK2A an adaptor of autophagy has two AIMs: a ubiquitin-like domain and a ubiquitin-associated domain. [102]. BIN2 kinase regulates DSK2 binding to ATG8, phosphorylating DSK2 around the AIM domains to improve its capacity to bind with ATG8 [102]. However, whether BES1 is damaged before or after entering the nucleus is unclear.

In mammals, nucleophagic activity is associated with genotoxic and oncogenic stress [101]. Although pathogenic conditions trigger nucleophagy in mammalian cells, the Nem1/Spo7–Pah1 axis and the orthologous CTDNEP1/NEP1R1-lipin complex are conserved from yeast to mammals. Similarly to its counterpart, the CTDNEP1/NEP1R1-lipin complex is found in the nuclear envelope. [23]. Nucleophagy in *S. cerevisiae* is mediated by the autophagic cargo receptor Atg39, which is found on the outer nuclear membrane [103]. Atg39 interacts with Atg8 via AIM within its cytosolic N-terminal region and subsequently interacts with the cargo receptor adaptor Atg11 via an Atg11 binding region (11-BR) (Figure 2c). Both of these interactions with Atg11 and Atg8 are essential for macronucleophagy [103]. However, future research is needed to determine whether nucleophagy occurs in mammalian cells under physiological circumstances.

### 3.4. Ribophagy

The selective autophagy of ribosomes can be observed in plant cells in addition to the above-mentioned particular autophagy pathway: For instance, a selective autophagy mechanism relying on ATG5 has been found in *Arabidopsis* and is involved in rRNA turnover [104]. It was recently observed that NUFIP1 is a ribophagy receptor in mammals that is essential for ribosome selective degradation during starvation (Figure 2) [105]. *Arabidopsis* has a homolog of mammalian NUFIP1; however, more research is required to determine whether *Arabidopsis* NUFIP1 is likewise engaged in ribophagy (Figure 2d) [106]. A new class of ATG8 interactors with a Ubiquitin-interacting motif (UIM)-like domain interacts with ATG8 in yeast/plants and animals has just been characterized [95]. As a result, additional selective autophagy routes are likely to be uncovered soon. Plant cells can efficiently eliminate damaged or unwanted cell components through these diverse types of selective autophagy pathways, ensuring plant survival and cell viability during environmental constraints.

### 3.5. Lipophagy

Lipids in membrane organelles serve as energy generation substrates as well as cellular structural materials. Fatty acids are first stored as triacylglycerol (TAG) in the lipid droplets (LDs) before being used directly for *β*-oxidation [107,108,109]. Lipolysis breaks down LDs into fatty acids for the cell caused by lipophagy, a selective autophagy mechanism found in mammalian cells [110,111]. Plant lipophagy processes were less studied than yeast’s and mammals’ [112,113]. In rice, LDs carrying TAGs in the tapetum are required during pollen maturation as a source of lipid components [114]. LDs encased in vacuoles have been discovered in rice tapetum cells, and LD-like structures were found in greater abundance in the cytoplasm of *Osatg7* and *Osatg9* mutants than in the wild type, showing that LDs in plants may also be degraded by lipophagy [114]. Furthermore, lipidomic research revealed that these mutant anthers had impaired phosphatidylcholine (PC) editing and lipid desaturation during pollen maturation, demonstrating that autophagy is involved in regulating lipid metabolism during plant development [114,115]. Under normal and limiting conditions in Arabidopsis, for the synthesis of TAG, organelles’ autophagy can offer a source of fatty acids, demonstrating that autophagy could increase TAG synthesis. A lipase sugar-dependent 1 (SDP1), responsible for the initiation of catabolism of TAG, hydrolyzes TAGs that are stored in LDs under normal conditions [116]. However, lipophagy, on the other hand, is driven by nutritional deprivation and causes the LDs to be degraded for energy production [109]. In the *atg5* mutant, for the synthesis of fatty acid and beta-oxidation, the ER and peroxisomal proteins are upregulated, and the concentrations of phospholipids, galactolipids, and sphingolipids are altered, suggesting that lipid metabolism is adversely affected in mutant autophagy, which could affect plant lipid metabolism in addition to regulating the synthesis of TAG and the degradation of LD [117].

Lipophagy in mammals activates with the autophagosomal membrane recognizing cargo by interacting with light chain 3 (LC3) [118]. Through the interaction with ATGL’s LIR domain, LC3 stimulates the translocation of cytoplasmic ATGL to the LD and causes lipophagy, and by the activity of SIRT1 action, ATGL enhances lipophagy to regulate hepatic LD catabolism [119]. Lipases found in LD, such as PNPLA5 (patatin-like phospholipase domain-containing enzyme 5) have been linked to lipophagy and autophagic proteolysis [120]. In a mouse model with a high-fat diet, another lipase from the same family, PNPLA8, can similarly interact with LC3 to induce lipophagy. These lipases are vital in initiating lipophagy by promoting the recruitment of triglycerides and sterol esters, which directly contribute to the production of autophagosomes, in addition to their role in LD detection [121]. Furthermore, in deprived human hepatocytes, PNPLA3 (patatin-like phospholipase domain-containing enzyme 3) is required to produce autophagosomes during the lipophagy process (Figure 2e) [122]. Surprisingly, a forced lipophagy system based on a fusion of the LD-binding domain and p62 has been shown to diminish the number of LDs, lower the level of TG throughout embryonic development, and finally, cause developmental retardation in mouse embryos. Furthermore, lipophagy-induced embryos are resistant to lipotoxicity and indicate the elimination of excess LD [123].

### 3.6. ER-Phagy (Reticulophagy)

The endoplasmic (ER) reticulum is a network of membrane tubules that is significant for protein and lipid synthesis in the cytoplasm and for storing calcium. When unfolded, proteins accumulate in the ER, and the ER-associated degradation (ERAD) and the unfolded protein response (UPR) pathways are triggered [110]. UPR is a signaling pathway that aims to reduce the accumulation of misfolded proteins in organelles while enhancing their folding capacity [110]. ERAD, on the other hand, identifies misfolded proteins and translocates them to the cytoplasm for degradation by ubiquitin proteasome system (UPS) [124]. Furthermore, autophagy is triggered by ER stress, and autophagosomes generated during this time have been found to contain ER components [125]. The ER autophagy or reticulophagy helps to maintain cell homeostasis by counteracting ER enlargement during the UPR. In addition to ER stress, other stimuli have been proven to induce ER-phagy as well [125]. ER-phagy, similar to other types of selective autophagy, involves receptor proteins that play key roles in the selection of targets. In yeast *S. cerevisiae,* Atg39 and Atg40 mediate ER-phagy, where they localize to different domains of the ER and enable the production of autophagosomes by interacting with Atg8 [103]. In mammals, the family with sequence similarity 134 member B (FAM134B) protein is Atg40’s functional homolog with the conserved LIR motif and positive ER fragments co-localizing with LC3B. Furthermore, whereas FAM134B downregulation causes ER enlargement, its overexpression causes ER fragmentation and lysosomal degradation [126]. Both the reticulon domain and the LIR motif of FAM134B are essential for ER-phagy (Figure 2f). The recently identified soluble members C53, CALCOCO1 (identified for homology with the xeno-phagy receptors TAXBP1 and CALCOCO2), and Sequestosome1/p62 extended the list of mammalian ER-phagy receptors [127,128,129]. Finally, the ER stress sensor IRE1a and two cytosolic autophagy receptors with a ubiquitin-binding domain, NBR1, and optineurin, have been involved in ER turnover and polypeptide clearance from the ER membrane [130].

The *Arabidopsis thaliana* Atg8-interacting proteins ATI1 and ATI2 were the first ER-phagy receptors reported in plants (Figure 2f) [131]. They lack homologs in yeast and higher eukaryotes and feature a single transmembrane domain and Atg8 interacting motif (AIM) in their cytosolic N-terminus and were found in the ER under favorable conditions. Carbon deprivation segregates ATI1 and ATI2 in the ER network into spherical entities that are subsequently transported to the vacuole after interacting with Atg8 [131]. The ER membrane proteins AtSec62 (*A. thaliana*), the reticulon homology domain (RHD)-containing proteins RTN1 and RTN2 (*Zea mays*), and the soluble protein Atc53 are all members of the ER-phagy receptor family in plants (*A. thaliana*) (Figure 2f) [128].

### 3.7. Mitophagy

Although several mechanisms mentioned above are essentially similar in plants, mitophagy regulators are considerably different in yeast/animals and plants. Mitophagy is the term for autophagic selective degradation of mitochondria. Autophagy is responsible for removing mitochondria, whether owing to injury, altered energy demands, or controlled cell maturation, as in the case of reticulocytes’ loss of mitochondria. Mitophagy is induced by various stimuli that cause mitochondrial damage, including hypoxia, chemical uncouplers, and reactive oxygen species (ROS) [132]. Additionally, mitophagy can occur in mammalian reticulocytes and the enterocyte cells of the Drosophila intestinal midgut in response to developmentally controlled alterations in the cell [133]. Moreover, during *C. elegans* development, mitophagy is also required to remove paternal mitochondria from fertilized oocytes [10]. Pink1 and *Parkin* genes, linked to familial Parkinson’s disease, are the most well-studied mitophagy pathways [134]. PINK1 phosphorylates a variety of targets, including ubiquitin and recruiting and activating Parkin, an E3 ubiquitin ligase [135]. Parkin then works as an amplifier of the mitophagy signal provided by PINK1. These ubiquitinating mitochondrial surface proteins can be detected by cargo receptor proteins, which transport mitochondria to autophagosomes for degradation [136]. Multiple receptors, including p62/SQSTM1, NIX/BNIP3L, BNIP3, FUNDC1, NDP52 (CALCOCO2), TAX1BP1, and optineurin (OPTN), have been involved in mitophagy in mammals [135].

Mitophagy appears to have a role in development, senescence, stress response, and programmed cell death (PCD) in plants [137]. On the other hand, plants lack many of the genes that drive mitophagy in yeast and animal cells, and no plant proteins that identify defunct mitochondria for autophagic degradation have yet been discovered. In plants, chloroplasts co-exist in energy generation alongside mitochondria, and chloroplasts are targeted by autophagy via a process called chlorophagy [138]. An early study indicated that mitochondria were encased with a double membrane structure similar to ER in mung bean (*Vigna radiata*) during autophagy [139]. Notably, these mitochondrial autophagous structures have been found to combine with lytic vacuoles. Recently, mitochondrial proteins and vesicles were shown to be degraded by autophagy in Arabidopsis during senescence [25]. A homolog of yeast ATG11 (and mammalian FIP200/RB1CC1) has recently been discovered in Arabidopsis. It is involved in mitophagy in nitrogen-depleted circumstances [25,64]. During senescence-induced mitophagy, ATG7, an E1-like enzyme, is also important as it facilitates the conjugation of ATG8 with phosphatidylethanolamine (PE) and ATG12 with ATG5, resulting in ATG8–PE and ATG5–ATG12 complexes, respectively [25,76].

### 3.8. Pexophagy

Peroxisomes are small round organelles surrounded by a single lipid bilayer present in most eukaryotes [140]. Despite their morphological similarity and conserved functions in all eukaryotes, major differences in peroxisomes have been found between plants and animals [141]. The selective autophagy pathways in eukaryotes require specific cargo receptor(s) and/or adaptors. Two kinds of pexophagy cargo receptors have been described in yeast and mammals, which differ in their capacities to bind ubiquitylated cargos [142]. In yeast, two AIM-containing pexophagy receptors (Atg30 and Atg36) become attached to peroxisome surface proteins such as Pex3, Pex5, or Pex14 (33), and then the Atg30 and Atg36 recruit the autophagic machinery by interacting with Atg8 and Atg11 [143]. Mammals do not have Atg30 or Atg36 but instead use p62/SQSTM1 or NBR1 as pexophagy adaptors that bind ubiquitylated forms of PEX5 or PMP70 (Figure 2g) [144].

Although plant peroxisome proteins are targets of ubiquitylation, plants do not have obvious orthologs of either Atg30 or Atg36, and there is no direct evidence that plant NBR1 is the pexophagy receptor, even though the co-localization of NBR1 and ATG8 in electron-dense peroxisomal cores in Arabidopsis plants exposed to Cd has recently been reported [145]. However, the peroxisome proteins PEX6 and PEX10 in *Arabidopsis* were recently shown to interact with ATG8 via their AIMs, suggesting that they may be the potential receptor for driving pexophagy in plants (Figure 2g) [13]. In addition, by using forward genetic screening, the peroxisomal matrix protease LON 2 was identified, mutation of which consistently recovers Arabidopsis *atg* mutants. Notably, the autophagy of *lon2* peroxisomes does not require NBR1, but NBR1 may play an important role in LON2-independent pexophagy [146]. Collectively, it is still worthy to explore if the ubiquitination of PEXs, such as PEX3, PEX5, and PEX14 reported in yeast and mammals, is also involved in plant pexophagy [147].

### 3.9. Lysophagy

The lysosome, a membrane-bound acidic organelle is required for eliminating unwanted intracellular compounds. The lysosome contains a significant number of hydrolytic enzymes that are involved in degradation. The lysosome’s destabilization and the leakage of these hydrolytic enzymes are detrimental to the cell [148]. Furthermore, if damaged lysosomes are not removed, the intracellular lysosome’s number remains unchanged, and cells are incapable of sustaining cellular homeostasis. As a result, to keep cellular homeostasis, the cell uses an autophagic mechanism called lysophagy [149,150]. Lysophagy can be triggered by several factors that cause lysosomal degradation (Figure 2h). Photochemical internalization is a method that enhances gene transport by light-induced lysosome breakdown that has been used to activate lysophagy in the lab [149]. Mineral crystals such as monosodium urate and silica, viral or bacterial toxins, lysosomotropic chemicals, lipids, and proteases have all been shown to disrupt lysosomal membranes in vivo [148,150]. In the case of lysosomal damage, ubiquitination coincides with the vigorous employment of the autophagy receptor SQSTM1/p62 that is essential for efficient lysophagy (Figure 2h). Although research into the mechanisms that regulate lysophagy is incomplete, it has been revealed that, following lysosomal injury, LC3 and galectin-3 are employed to the wounded lysosome [150]. In mouse embryonic fibroblasts (MEFs), damaged lysosomal membranes are galectin-3 positive, ubiquitinated, and co-localize with p62 [150]. Furthermore, in HeLa cells, a similar connection between p62 and ubiquitin has been linked with damaged lysosomes [149]. These findings point out to ubiquitination and subsequent recruitment of the cargo adapter protein p62 in this mechanism. There are still many unanswered questions about the molecular aspects of lysophagy. However, biochemical and functional studies of ubiquitin, ubiquitin receptors, and the factors that affect their activity could help us better understand this process.

## 4. Chlorophagy

Plants and photoautotrophs have chloroplasts that are responsible for photosynthesis and are essential for the metabolism. Despite the fact that plants are sessile, cellular components must be used and recycled to survive and thrive in a variety of conditions. After the degradation of cellular macromolecules, their components are mobilized and reused during plant senescence. For instance, the degradation of chloroplast proteins such as Rubisco (ribulose-1, 5-bisphosphate carboxylase/oxygenase) is a significant source of nitrogen.

In senescing leaves, the primary method for chloroplast protein degradation was sequential breakdown within the vacuole known as chlorophagy [151]. The chloroplast, similar to the nucleus, can be digested piecemeal or through complete organelle autophagy. Senescence-associated vacuoles (SAVs) and Rubisco-containing bodies (RCBs) can cause piecemeal chloroplast degradation (Figure 2i). RCBs are Rubisco and Gln synthetase-containing double-membrane entities produced from the chloroplast envelope. Furthermore, the RCB is then encircled by various membrane structures, including the isolation membrane in the cytoplasm, after pinching off from the chloroplast.

Damaged chloroplasts are ubiquitylated by PUB4 (Plant U-BOX Protein 4), the cytosolic ubiquitin ligase as part of the whole chloroplast process. The ubiquitylated chloroplasts are then encapsulated and transported to the vacuole via ATG8-decorated autophagic vesicles [152]. Autophagy mediated by RCB has been demonstrated to be dependent on Atg4 and Atg5, and is essential for the recycling of protein during abiotic stresses [153,154]. The RCBs are formed by the fission of stroma-filled tubules protruding from the chloroplast, a process in which the ESCRT component CHMP1 (Charged Multivesicular Body 1) may play a key role, and they are degraded in the vacuole by autophagy [13]. SAVs have also been linked to chloroplast autophagy on a piecemeal basis. These tiny and lytic vacuoles are only present in senescing tissues and contain stromal proteins, including Rubisco and Gln synthetase, similar to RCBs. Unlike RCBs, they do contain chlorophyll *a* and do not appear to employ any autophagic machinery. The decline in chloroplast number is hampered in *atg4* mutants [155], suggesting that there may be an *ATGs*-dependent selective autophagy of the chloroplast. Recently, plant-specific proteins ATG8-interacting protein 1 and 2 (ATI1 and ATI2), which are found in the plastid-derived bodies and ER, are proposed to be involved in *ATGs*-mediated chlorophagy (Figure 2i) [156]. Plastid proteins from the outer envelope are translocated to the vacuole via the ATI1-decorated plastid structures (ATI1-PS)-mediated autophagy [156]. Notably, incomplete degraded chloroplasts in the vacuole were also found in plastid protein Tic40 (*ppi40*) mutants of *Arabidopsis* [157]. Because these defects were observed in a starvation-independent way, it is assumed that plants could use the autophagy-independent pathway to eliminate chloroplasts for quality control. This possibility was recently validated by the characterization of the Chloroplast Vesiculation protein (CV)-containing vesicles (CCVs) pathway [158].

In summary, the mechanisms underpinning chlorophagy are still poorly understood, leaving many open questions. For instance, it is unclear when and how cargo receptor proteins target the entire chloroplast to trigger chlorophagy. Although plants have putative cargo receptors of autophagy, such as ATI1 and ATI2 [131], as well as RCB receptors, it is still unclear how these proteins are specifically assigned in operating chlorophagy, and thus, novel approaches and/or components are needed.

## 5. Advances of Selective Autophagy in Plant

Plant selective autophagy research is progressing at a rapid pace. The majority of the previously documented selective autophagy pathways in metazoans have lately been discovered to work in plants as well [159,160].

Plants have an NBR1-like protein that is necessary for autophagosome degradation of ubiquitinated peroxisomes [161]. Peroxisomes are ubiquitous organelles in plants that control numerous metabolic events such as photorespiration, fatty acid β-oxidation, and the glyoxylate cycle [162]. Reactive oxygen species (ROS) are primarily produced by peroxisomes, making them prone to oxidative damage. Multiple antioxidative enzymes are found in the peroxisomes to eliminate excessive ROS, such as catalase, which degrades H_2_O_2_ exclusively [163]. High glucose levels of wild-type plant roots cause the accumulation of ROS. On one side, reactive oxygen species (ROS) oxidize active IAA (indole3-acetic acid) and constitutive pexophagy, on the other hand, it is aided by the increased level of ROS, which reduces root meristem activity by inhibiting IAA production [164]. However, autophagy deficit in *atg5* and *atg7* affects the transmission of the high glucose signal to the peroxisomes, increasing IAA and root meristem activity and resulting in increased primary roots compared to wild type under enhanced glucose conditions [164]. Recently, Arabidopsis plants exposed to Cd were found to have NBR1 and ATG8 co-localized in electron-dense peroxisomal cores [145]. Peroxisome oxidation and pexophagy were induced by Cd exposure, however, the *Arabidopsis* mutant *rbohC* (NADPH oxidase C) and *gox2* (glycolate oxidase 2) inhibit this process significantly by reducing ROS generation in Arabidopsis. Pexophagy is a key component of quick plant responses to Cd (cadmium), as it protects peroxisomal populations and the cell redox homeostasis [145].

In the case of chlorophagy, ESCRT III component CHMP1 proteins are involved in the efficient recycling of fragmented chlorophagy vesicles containing stromal proteins, according to an exquisite cell biology investigation [165]. Unlike chloroplasts related chlorophagy, little is known mechanistically about plant mitophagy even though accumulating genetic and cytological evidences suggest that mitochondria are recycled through autophagy [25], because some of the known mitophagy receptors and regulators are absent in plants [137]. It is also indistinct whether chlorophagy and mitophagy have any similarities, such as if they would share the same autophagy receptor. As a case, the ATI1/2 may service as receptor for both chlorophagy and ER-phagy [134,156]. Additionally, if plants, such as metazoans, utilize piecemeal mitophagy mechanisms is yet to be identified.

Moreover, mitophagy has been discovered in plants, where it is involved in development, stress response, senescence, and PCD [137]. The relationship between mitophagy and senescence is well-known, although its mechanistic understanding is lacking despite the fact that the core ATG proteins are well conserved in plants and are necessary for the senescence-induced degradation of mitochondrial vesicles and mitochondria-resident proteins [64]. Arabidopsis has a number of mitochondrial membrane proteins with ATG8-interacting motifs, according to a bioinformatic investigation [166], which are thought to be mitophagy receptors [137]. *Arabidopsis* mutants lacking key autophagy components are more vulnerable to UVB exposure, resulting in increased leaf chlorosis [152], demonstrating that autophagy is critical for cellular homeostasis in response of UVB. The number of mitochondria in wild-type leaves falls in response to UVB exposure but increases in *atg* (*atg2*, *atg5*, and *atg7*) mutants [138]. Notably, following UVB damage, confocal and electron microscopy observations reveal that *atg5* and *atg7* mutant plants accumulate fragmented and tiny mitochondria in the cytoplasm [138]. A substantial percentage of mitochondria in UVB-damaged *atg* leaves fail to collect tetramethylrhodamine ethyl ester (TMRE) [138], suggesting that damaged mitochondria stay in the mutant cytoplasm and their membranes are depolarized in response to UVB damage. Additionally, a variety of evolutionary conserved mitochondrion-associated proteins are also involved in the quality control of mitochondria [167]. Notably, the clustered mitochondria protein (CLU) is essential for mitochondria distribution and function in yeast, plants, and animals [168]. Mitochondrial membrane potential is abolished when uncouplers such as 2,4-dinitrophenol (DNP), carbonyl cyanide, and p-trifluoro-methoxyphenylhydrazone (FCCP) are applied. More recently, in Arabidopsis roots, mitophagy eliminates depolarized mitochondria in response to uncoupler treatment [169]. These findings support the idea that plant mitophagy plays a critical role in mitochondrial quality control. Upon uncoupler treatment, friendly mitochondria (FMT) labeled with YFP is recruited to mitochondria and co-localizes with mCherry ATG8 [169]. Moreover, in *fmt* mutants, the uncoupler-induced mitochondrial degradation was reduced [169], suggesting that FMT has a direct role in mitophagy activation. In terrestrial plants, the shape and volume of mitochondria vary dramatically throughout reproductive development [170]. The tapetum is the anther’s innermost layer, which supplies nutrition to pollen grains as they mature, and later undergoes programmed cell death (PCD) [171]. Mitochondrial fragmentation and a reduction in overall mitochondrial volume occur before PCD in Arabidopsis tapetal cells [170]. Previously, it was observed that autophagy is necessary for the regulated PCD of tapetal cells in rice [114]. As a result, autophagy could be involved in mitochondrial degradation during PCD of tapetal cells.

## 6. Conclusions and Future Perspective

Over the last few decades, autophagy research has progressed to unprecedented depths, with studies on the interaction and communication between autophagosomes and other organelles. Many of the protein components and molecular mechanisms involved in autophagy have been identified and key regulatory factors have also been discovered, such as the TOR complex [172] and SnRK1 [173]. Studies on the roles of autophagy upon various stress have enabled us to understand the potential contributions of autophagy to crop breeding. However, several important open questions about the underlying molecular mechanisms of autophagy still remain to be further investigated, including the identification of specific SARs for certain types of organelles’ selective autophagy, possible crosstalk between autophagy and other regulatory pathways (ubiquitin-proteasome etc.), and the manipulation of *ATGs* or autophagic machinery for robust improvement of crop yield and therapy of human diseases.

It is no doubt that the rapidly expanding collection of SARs and cargo proteins by high throughput screening of ATG8-interacting proteins would extend our knowledge of the multiple roles of autophagy in organism development and growth, as well as their response to stress. However, it is still difficult to precisely determine the specific SARs and/or cargo proteins due to the greater diversity of gene families and functions in both plants and animals. Moreover, as mentioned above, the evolutionary divergence of certain SARs derived from animals or plants may also impose restrictions on the identification of these SAR homologs in plants or animals. For example, detailed studies on the detection of ubiquitinated cargo by mammalian p62 have been published, while similar attempts in plants have not been as successful. On the other hand, the employed experimental approaches are currently limited, and thus specific genetic screening is still desirable, such as suppressors or enhancers screening of *atg* mutants or subcellular localization of fluorescence-labled ATGs.

To summarize, despite the fact that there is still much work to be done, autophagy investigations are nonetheless exciting and relevant since they have the potential to target virtually all organelles for degradation, thereby facilitating the quality control of organelles upon various stresses. With further research and the application of new methodologies, we will undoubtedly obtain a better knowledge of the autophagy interaction network, as well as the extensive insights into the conserved and distinct mechanisms of autophagy between plants and animals.

## Figures and Tables

**Table 1:** Identified ATGs genes in yeast, mammals, and plants.

Yeast	Mammalian	Plants	Function	Reference
ATG1	ULK1, ULK2	AtATG1a-1c,1t, OsATG1a-1d	Protein kinase; functions in the induction of autophagy	[24,25,26]
ATG13/APG13	ATG13	AtATG13a-13b, OsATG13a-13c	Phosphorylated by TORC1; forms complex with ATG1 to function in the induction of autophagy	[25,26,27]
ATG17	FIP200	Not identified	Essential for both stability and phosphorylation of ULK1	[25,28]
ATG29	Not identified	Not identified	Function in induction and regulation of autophagy	[25,29]
ATG31	Not identified	Not identified	Function in induction and regulation of autophagy	[30]
ATG9/APG9/AUT9/CVT7	ATG9A, ATG9B	AtATG9a, OsATG9a-9b	Membrane protein; deliver membrane to the forming autophagosome	[31,32]
ATG2		AtATG2a, OsATG2a	Atg18-interacting protein; function in autophagosome formation	[32,33]
ATG18/AUT10/CVT18	WIPI-1, 2, 3, 4	AtATG18a-18h, OsATG18a-18f	PI(3)P-binding protein; involved in the formation of autophagosome	[32,33,34]
ATG27	Not identified	Not identified	Protein required for autophagy-dependent cycling of Atg9	[35]
ATG6/VPS30/APG6	BECN1	AtATG6a, OsATG6a	Beclin1 (the core subunits), bcl2-interacting protein; functions in nucleation	[36,37,38]
ATG14	ATG14	AtATG14a-14b	Enhancer of autophagosome formation; function in nucleation	[37,39,40]
ATG12/APG12	ATG12	AtATG12a-12b	Ubiquitin-like, conjugates to Atg5; function in autophagosome membrane expansion	[41,42]
ATG5/APG5	ATG5	AtATG5a	Ubiquitin-like ligase, conjugated by Atg12	[42,43]
ATG16	ATG16L1	AtATG16L	interacts with Atg5; stimulate ATG8–PE conjugation reaction	[44,45]
ATG7/APG7	ATG7	AtATG7a, OsATG7	E1-like enzyme for Atg12 and Atg8/LC3 conjugation	[41,46]
ATG10/APG10	ATG10	AtATG10a	E2-like enzyme covalently conjugates Atg12 to ATG5	[41,47,48]
ATG8/APG8/AUT7	MPA1LC3B/LC3B	AtATG8a-8i, OsATG8a-8e	Ubiquitin-like conjugates to PE	[41,49,50]
ATG3/APG3	ATG3/APG3	AtATG3	Function as E2-like enzyme for Atg12 and Atg8/LC3 conjugation	[51,52]
ATG4/APG4/AUT2	ATG4A-D	AtATG4a-4b	Cytosolic cysteine protease for processing and recycling of Atg8/LC3	[32,53]

## Data Availability

The data presented in this study are available in review.

## Abbreviations

AIM	Autophagy-interacting motif
AMPK	AMP-activated protein kinase
ATG	autophagy-related
CAMKK2/CaMKKβ	calcium/calmodulin-dependent protein kinase kinase 2, beta
CHMP1	Charged Multivesicular Body 1
CMA	chaperone-mediated autophagy
Deptor	DEP domain-containing mTOR interacting protein
ERAD	endoplasmic reticulum associated degradation
FREE1	FYVE domain protein necessary for endosomal sorting 1
FYCO1	FYVE and coiled-coil domain containing 1
HOPS	homotypic vacuole fusion and protein sorting
LC3	light chain 3
PAS	phagophore assembly site
PC	phosphatidylcholine
PCD	programmed cell death
PE	phosphatidylethanolamine
PUB4	Plant U-BOX Protein 4
RAPTOR	regulatory-associated protein of TOR
RCBs	Rubisco-containing bodies
RHD	reticulon homology domain
ROS	reactive oxygen species
SAV	Senescence-associated vacuoles
SnRK1	Sucrose nonfermenting-1-Related protein Kinase 1
TMRE	tetramethylrhodamine ethyl ester
TOR	target of Rapamycin
ULK	unc-51-like kinase
UPR	unfolded protein response
UPS	ubiquitin proteasome system
XPO1	export-dependent process mediated by exportin 1
